# The Changing Epidemiology of Kunjin Virus in Australia

**DOI:** 10.3390/ijerph10126255

**Published:** 2013-11-25

**Authors:** Natalie A. Prow

**Affiliations:** 1 Australian Infectious Diseases Research Centre, University of Queensland, St Lucia, QLD, 4072, Australia; E-Mail: n.prow@uq.edu.au; Tel.: +61-7-3365-4648; Fax: +61-7-3365-4620; 2 School of Chemistry and Molecular Biosciences, University of Queensland, St Lucia, QLD, 4072, Australia

**Keywords:** West Nile virus, Kunjin virus, epidemiology, Australia

## Abstract

West Nile virus (WNV) is a mosquito-borne virus responsible for outbreaks of viral encephalitis in humans and horses, with particularly virulent strains causing recent outbreaks of disease in Eastern Europe, the Middle East and North America. A strain of WNV, Kunjin (WNV_KUN_), is endemic in northern Australia and infection with this virus is generally asymptomatic. However in early 2011, an unprecedented outbreak of encephalitis in horses occurred in south-eastern Australia, resulting in mortality in approximately 10%–15% of infected horses. A WNV-like virus (WNV_NSW2011_) was isolated and found to be most closely related to the indigenous WNV_KUN_, rather than other exotic WNV strains. Furthermore, at least two amino acid changes associated with increased virulence of the North American New York 99 strain (WNV_NY99_) compared to the prototype WNV_KUN_ were present in the WNV_NSW2011_ sequence. This review summarizes our current understanding of WNV_KUN_ and how the epidemiology and ecology of this virus has changed. Analysis of virulence determinants of contemporary WNV_KUN_ isolates will provide clues on where virulent strains have emerged in Australia. A better understanding of the changing ecology and epidemiology associated with the emergence of virulent strains is essential to prepare for future outbreaks of WNV disease in Australia.

## 1. Introduction

West Nile virus (WNV) has emerged as a global public health concern, causing large outbreaks in the Americas, Europe and more recently Australia [1,2,3,4,5,6,7,8,9]. Kunjin virus (WNV_KUN_) is classified within a clade of the WNV group and has traditionally been associated with mild and rare disease in humans and horses in Australia. Phylogenetic analysis has suggested that WNV emerged in Africa and can be separated into at least seven genetic lineages [10]. There are two main lineages (I and II), with lineage I containing WNV_KUN_ isolates and WNV isolates from North, West and Central Africa; southern and eastern Europe; the Middle East; and New York. Lineage I can be further divided into clade 1a containing WNV isolates from around the world and the Australian WNV_KUN_ isolates forming clade 1b [11].

Following extensive flooding across Eastern Australia in 2011 promoting ideal conditions for freshwater mosquito breeding, an unprecedented outbreak of equine encephalitis occurred, leading to the isolation of the first virulent strain of WNV_KUN_ to cause a major outbreak, designated WNV_NSW2011_ [2]. For clear distinction throughout this review, WNV_NSW2011_ will be used to describe the virus isolated from New South Wales (NSW) during the 2011 Australian outbreak, and WNV_KUN_ will refer to all other isolates, unless specifically stated otherwise. Prior to this outbreak, there were two Australian WNV_KUN_ equine isolates, Boort and 35911. The Boort strain was isolated from the cervical spinal cord of a horse with encephalomyelitis in 1984 from Victoria (VIC), where a small outbreak of unusual equine nervous disease occurred. While other horses in this outbreak displayed similar symptoms, Ross River virus was isolated from a further two horses and these horses did not have detectable antibodies against WNV_KUN_ [12]. The 35911 strain was also isolated in 1984 from the Hunter Valley, NSW [13]. There has also been a single human isolate of WNV_KUN_, designated Hu6774, from a non-encephalitic patient [10].

This review will summarise what is currently known about the epidemiology and ecology of WNV_KUN_ in Australia and how these factors have recently changed. The outbreak in 2011 is an example of how a previously benign virus can evolve to emerge as a new public health threat. The emergence of this new virus has instigated additional studies into the virulence of contemporary strains of WNV_KUN_. Possible ecological factors that may have contributed to the unusual epidemiological features of the 2011 outbreak will also be proposed. For a more historical overview of WNV_KUN_, the reader is referred to extensive reviews by Hall *et al*., [14] and Marshall [15].

## 2. Ecology and Epidemiology

### 2.1. Vectors and Vertebrate Hosts

WNV_KUN_ is maintained in mosquito-bird transmission cycles, where mosquitoes act as primary vectors and birds as amplifying hosts. *Culex* (*Cx.*) *annulirostis*, a fresh water mosquito found across Australia, is the principal vector of WNV_KUN_ and Murray Valley encephalitis virus (MVEV) in Australia. MVEV co-circulates in the same geographic regions and utilizes the same vectors and vertebrate hosts as WNV_KUN._ MVEV remains the only proven cause of fatal human arboviral encephalitis in Australia [16], and causes sporadic outbreaks of encephalitis in humans, mainly in northern Australia. Major outbreaks of MVEV occurred in 1951, 1956, 1974 and in 2000 [17]. For a recent clinical review of MVEV in humans, the reader is referred to Knox *et al.*, [18]. MVEV also causes occasional cases of encephalitis in horses [19,20,21].

Since the first isolation of WNV_KUN_, the majority of isolates from field-caught mosquitoes have been from *Cx. annulirostris* [16,22]. WNV_KUN_ has also been occasionally isolated from other mosquito species including *Aedes* (*Ae.*) *tremulus*, *Cx. australicus*, *Cx. squamosus*, *Ae. alternans*, *Ae. nomenensis*, *Ae. vigilax*, *Anopheles amictus*, and *Cx. quinquefasciatus* [16,22,23]. Horses and humans are generally considered as dead-end hosts, as the resultant viraemia within these species is insufficient to allow further transmission of the virus. Wading birds (particularly *Nycticorax calendonicus*, the rufous night heron) are considered an important natural reservoir of WNV_KUN_. However, a range of birds and mammalian hosts can act as vertebrate hosts for WNV_KUN_ [15,24]. WNV_KUN_ is endemic in northern Australia and has been recorded periodically in south-east Australia through seroconversion in sentinel chickens and human infections [16].

### 2.2. Surveillance Using Sentinel Chickens

**Figure 1:**
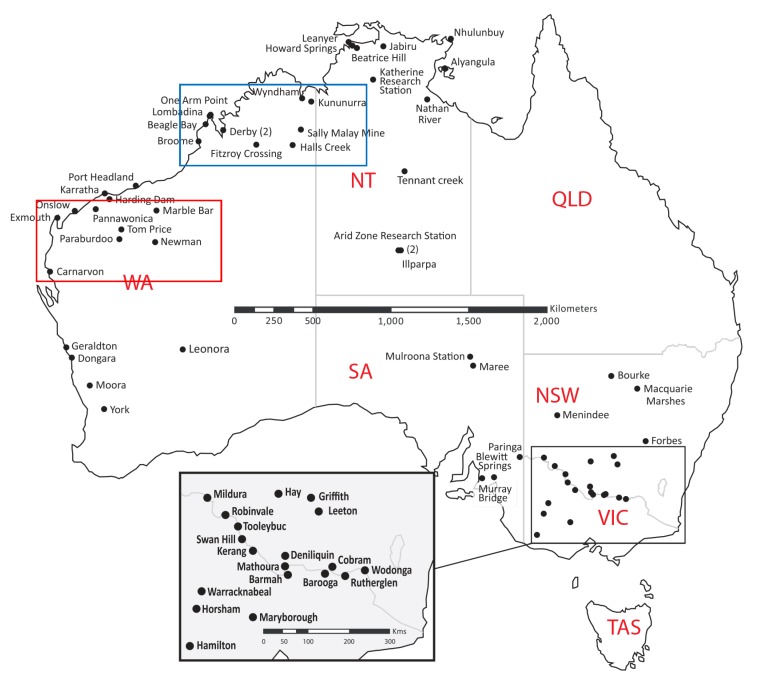
Location of sentinel chicken flocks around Australia, 2010–2011, Adapted from CDI Annual Report Vol. 37 No. 1 2013, used by permission of the Australian Government [26]. Australian states are shown in red. WA—Western Australia, NT—Northern Territory, QLD—Queensland, NSW—New South Wales, VIC—Victoria, TAS—Tasmania. Blue box represents the Kimberley region and the red box the Pilbara region. Inset represents sentinel chicken flocks in Victoria and New South Wales.

The National Arvbovirus and Malaria Advisory Committee (NAMAC) provide expert technical advice on arboviruses to the Australian Health Protection Principal Committee (AHPPC) through the Communicable Diseases Network of Australia (CDNA). NAMAC assists in the detection, management and control of outbreaks of arboviruses and provides advice on the risk of these diseases. The sentinel chicken program provides early warning of arboviral disease activity and allows NAMAC to provide timely warnings on arbovirus activity around Australia. For more details about the sentinel chicken program, NAMAC publish an annual report that can be located on the Australian Government Department of Health and Ageing website [25]. Flocks of sentinel are distributed throughout the Northern Territory (NT), NSW, South Australia (SA), VIC and Western Australia (WA). While the number and location of the flocks may change slightly from year to year, flocks are well distributed to provide valuable information about flavivirus activity in participating states (Figure 1). The program aims to provide early warning of the endemic arboviruses, MVEV and WNV_KUN_. This was evident in early 2011 when sentinel chickens in south-eastern Australia widely seroconverted to MVEV and WNV_KUN_ [26].

In WA, the sentinel chicken program allows the movements of both WNV_KUN_ and MVEV to be tracked [26]. Generally seroconversions are seen to both MVEV and WNV_KUN_ each year confirming that these viruses are endemic in northern WA [26,27,28]. Furthermore, there has been a trend of increasing numbers of seroconversions to WNV_KUN_ over several years (1996–2007) [29,30], followed by years with few seroconversions (2008–2009: 2 seroconversions; 2009–2010: 2 seroconverisons). Data from this program would suggest that MVEV is more active in the Kimberley region whereas WNV_KUN_ is more prevalent in the Pilbara region (Figure 1) [27].

Sentinel chickens can also provide a means to evaluate implemented control measures. The Ilparpa Swamp located in the NT (Figure 1), provides an ideal breeding site for *Cx. annulirostris* mosquitoes. A drainage system in the Ilparpa Swamp was established in early 2002 as a mosquito reduction method. Since this intervention there have been no seroconversions in sentinel chickens to MVEV or WNV_KUN_ and no human infections from these viruses in the NT [31], until the 2010–2011 wet season (November through March) [26].

Aside from data generated from sentinel chickens, some state agencies also use meterological surveillance data, such as the Southern Oscillation Index and rainfall deciles using Forbes and Nicholls models to assess the risk of outbreaks. The Southern Oscillation index is an important indication of climatic fluctuations over the Indian and Pacific Oceans and is closely related to Eastern and Northern Australian rainfall. Therefore this index has been used to develop methods for predicting rainfall fluctuations over Australia and their biological implications. Both the Forbes and Nicholls MVEV climatic models indicated probable activity in south-eastern Australia for the 2010–2011 season (typically the spring/summer months from September through to February) [26,32,33]. Additional studies analyzing remote sensing data are being evaluated for the development of rainfall-based predictive models. One such model using Multi-satellite Precipitation Analysis data provides a state-of-the-art data source for the development of a rainfall-based predictive model for flavivirus activity in tropical WA [34]. In addition to fluctuations in rainfall, changes in temperature are also likely to affect the transmission of WNV_KUN_. Distribution of mosquito species can be limited by temperatures, and thus tropical vectors are expected to move further south into currently cooler areas, when climate changes brings an increase in ambient temperature. Mosquitoes respond to local temperature increases in various ways and higher temperatures may lead to rapid development of larval populations, shorter times between bloodmeals, quicker incubation times for virus infections, and shorter life spans for adults [35]. Therefore, in a warmer climate, *Cx. annulirostris* mosquitoes might be expected to commence activity and reach high levels of abundance earlier, and maintain them longer; thus the season of arbovirus activity could be prolonged, with potentially greater levels of WNV_KUN_ transmission. However, while the predicted increases in temperature associated with climate change should provide better conditions for mosquito species; these populations will require concomitant increase in rainfall to ensure larval habitat and to maintain humidity for adult survival. Providing appropriate vertebrate hosts are nearby, climate change associated with increased rainfall and temperature could lead to increased WNV_KUN_ transmission [35].

### 2.2. Epidemiology of Human Infections

**Figure 2:**
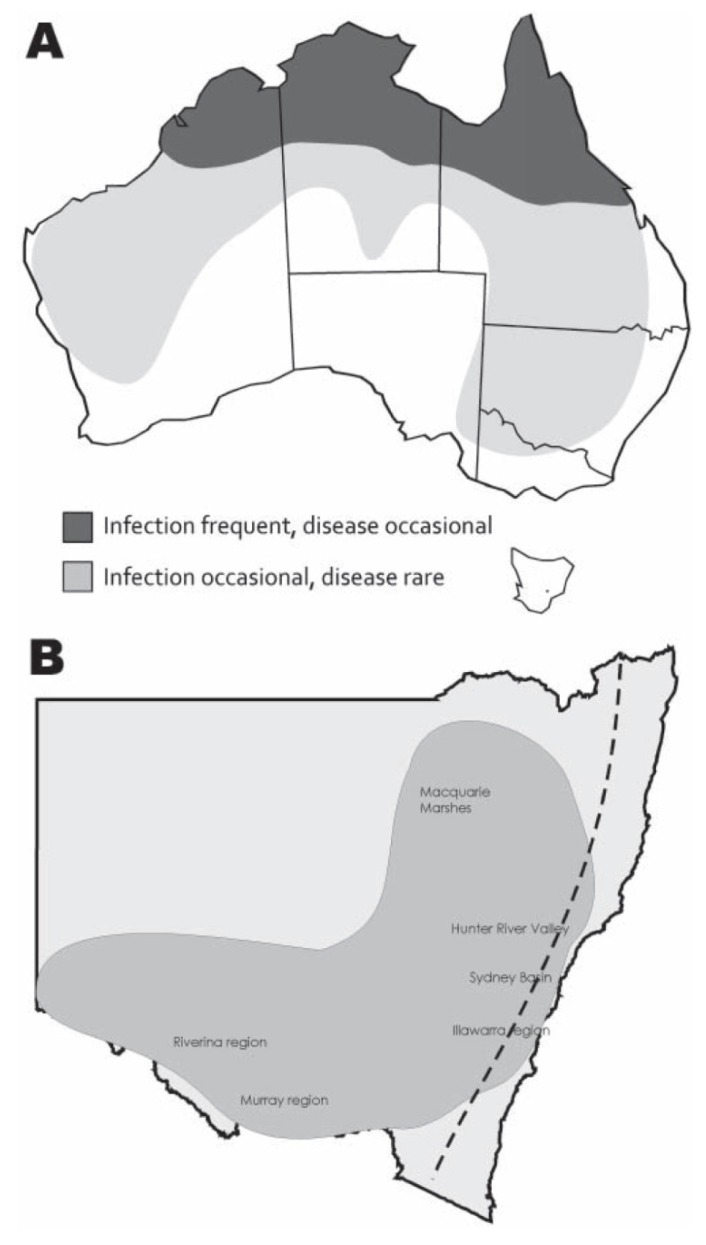
Historical distribution of WNV_KUN_ in Australia (**A**). Distribution of encephalitic horse cases in NSW during the 2011 outbreak (**B**). Dashed line indicates the Great Dividing Range. Adapted from [2].

While human infections with WNV_KUN_ are generally associated with mild symptoms, there has been a recent case that was associated with encephalitic symptoms [36]. A summary of the reported human cases and their location can be found in Table 1. The number of cases attributed to WNV_KUN_ has been lower than those reported for MVEV, except for 2004–2005 where four cases of WNV_KUN_ were reported. Even during the 2011 outbreak, only one mild human case of WNV_KUN_ was reported, despite over 1000 equine cases [2]. In comparison, 16 confirmed cases of MVEV were reported during 2011. In 2009–2010, 2 human cases of WNV_KUN_ were reported, with one of these cases exhibiting encephalitic symptoms [28,36]. In 2007–2008 there was one human case involving a male tourist from Israel. This represents the first report of a laboratory confirmed imported WNV infection in Australia. This person was likely infected in the Middle East where WNV is endemic [27,37]. In general, human cases of WNV_KUN_ are infrequent and mostly from northern Australia, with occasional infection and rare disease reported from southern regions of Australia (Figure 2, panel A).

**Table 1:** Number of confirmed cases of MVEV and WNV_KUN_ in Australia, 2004-2011.

Year	MVEV	WNV_KUN_
	Number of cases (location)	Number of cases (location)
2010–2011 *	16 (2: NSW; 2: NT; 2: SA; 9: WA)	1 (NT)
2009–2010 ^#^	0	2 (1: NT; 1: QLD)
2008–2009 ^€^	4 (2: NT; 2: WA)	3 (1: NT; 2: QLD)
2007–2008 ^$^	2 (1: NSW; 1: WA)	1 (VIC)
2006–2007 ^±^	0	0
2005–2006 ^≠^	1 (WA)	2 (WA)
2004–2005 ^§^	2 (1: NT; 1: QLD)	4 (3: QLD; 1: VIC)

* [26]; ^#^ [28]; ^€^ [38]; ^$^ [27]; ^±^ [29]; ^≠^ [30]; ^§^ [39]; NSW: New South Wales; NT: Northern Territory; QLD: Queensland; SA: South Australia; VIC: Victoria; WA: Western Australia.

## 3. Emergence of the First Virulent Strain in Australia to Cause an Outbreak

**Figure 3:**
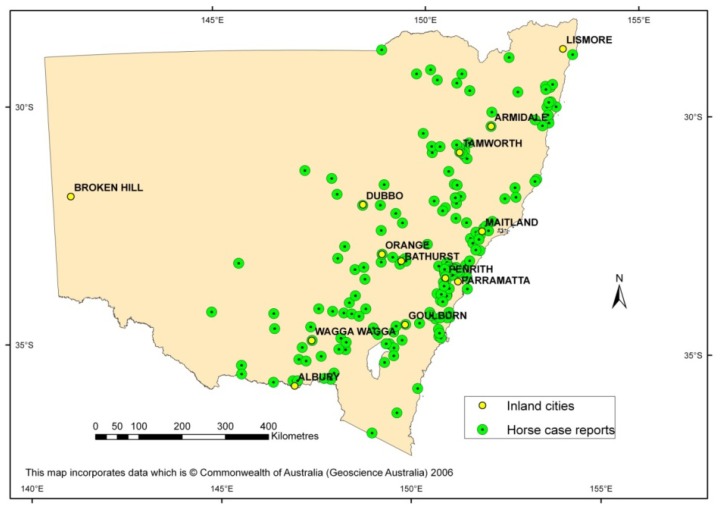
Distribution of horse cases during the 2011 outbreak. Adapted from [45].

The climatic conditions prior to 2011 for the NSW inland were above, to well above average rainfall for the entire second half of 2010, plus above average rainfall along the Murray River valley for the first quarter of 2011. The elevated rainfall led to increased vector populations with over 200,000 mosquitoes trapped at inland localities around NSW in 2011, being over six times that of the previous season [26]. Subsequently, a large number of arboviruses were isolated from NSW including seven WNV-like isolates from field-caught mosquitoes and one WNV-like virus from the brain of a deceased horse. All of these isolates were shown to be identical by sequence analysis and designated as WNV_NSW2011_, the virus responsible for the 2011 outbreak. Virus infections during this outbreak occurred in areas where WNV_KUN_ had not been seen previously, including major inland cities in NSW (Figure 3). During the 2011 outbreak there were over 1,000 equine cases and a mortality rate of approximately 10%–15% of infected horses [2]. Clinical signs of WNV_NSW2011_ in horses were typical of that described for WNV in North America and included changes in temperament, in-coordination, ataxia, weakness, muscle paralysis and tremors [40]. The horse-derived WNV_NSW2011_ isolate was found to be genetically most closely related to the indigenous WNV_KUN_, rather than other exotic WNV strains. However, at least two amino acid changes associated with increased virulence of WNV_NY99_ were present in the WNV_NSW2011_ sequence. Notably, WNV_NSW2011_ contained a glycosylated envelope protein at position 154 (N-Y-S) associated with neuroinvasion of WNV strains [41], as well as the phenylalanine residue at position 653 of the nonstructural protein 5 (NS5) that has been proposed to allow NS5 to be a potent antagonist of type I interferon-mediated JAK-STAT signaling [42]. However, the nonstructural protein 3 (NS3) motif, known to be responsible for increased bird virulence in WNV_NY99_ (Ala-Pro at amino acid 249) was not present in the WNV_NSW2011_ genome consistent with a lack of bird morbidity or mortality during the outbreak. A study by Bingham and colleagues have determined that a species of Australian Crow, the Little Raven (*Corvus mellori*), common in southern Australia was resistant to WNV infection, even with WNV_NY99_ [43]. Therefore, species differences between Australian and American crows contributing to differing susceptibility to WNV infection suggests that the change in the NS3 gene may have a limited effect on virulence to local avian species. However, additional experiments testing the susceptibility of other Australian corvid species are also required.

WNV_KUN_ isolates were obtained from equine and mosquito samples in NSW, VIC and SA and their genomes sequenced. WNV_KUN_ isolates from 2011 were almost identical with the Victorian and NSW isolates sharing 99% nucleotide identity [6]. Further analysis of WNV_KUN_ isolates from 2011 has identified a substitution within the NS5 gene at residue 49 (2577; I→V) located in the methyl transferase domain compared to the prototype WNV_KUN_ strain. This domain is involved in RNA capping and residue 49 forms part of the binding site for the monoclonal 5H1 in the αA3 motif [2,6,44]. Whether this change in the virus contributes to virulence or is simply an evolutionary marker remains to be elucidated.

Cases of encephalitic horses were located throughout most of NSW, west of the Great Dividing Range, but also extending through the Hunter River valley region, Sydney Basin, and Illawarra coastal region (Figure 2B). Cases also occurred in other southern Australian states, including VIC and SA. Based on serological findings, the causative agents were WNV_KUN_, MVEV and Ross River virus. In NSW, SA and WA, the majority of cases were due to WNV_KUN_ infections, whereas in VIC, Ross River virus comprised more than half of all confirmed cases [26]. The largest number of serologically positive samples specific for WNV_KUN_ came from NSW, while antibody positive samples were also identified from QLD, SA, VIC and WA. A large number of serological samples that were confirmed as flavivirus positive could not be clearly identified as MVEV or WNV_KUN_ (see Figure 1, [8]) due to inconclusive findings during the analysis. These results suggest a need for re-evaluation of current diagnostic tests for MVEV and WNV_KUN_ in the context of currently circulating virus strains.

Another interesting epidemiological feature of the WNV_NSW2011_ outbreak was the detection of WNV_KUN_ east of the Great Dividing Range in NSW. Indeed, WNV_NSW2011_ was detected despite relatively small mosquito populations in many of these areas, suggesting that the virus was transmitted more efficiently between mosquito vectors and mammalian hosts. Despite the abundance of WNV_KUN_ isolates from *Cx. annulirostris* mosquitoes in the past, it has been demonstrated that this mosquito has a relatively poor transmission rate in the laboratory [46]. Therefore, it has been suggested that the ability of this species to reach high population densities under optimal conditions deems it the primary WNV_KUN_ vector in Australia [14]. In light of relatively small mosquito populations in major urban areas during the 2011 outbreak, it seems likely that other mosquito species aside from *Cx. annulirostris* contributed to transmission of WNV_NSW2011_. Furthermore, a previous study by Jansen *et al.*, demonstrated that while *Cx. annulirostis* gave a overall high level of vector competence for WNV_NY99_; there was intraspecific variation between populations, with mosquitoes collected from Brisbane (south-east QLD) appearing to be less efficient vectors compared to populations collected from Sydney (NSW) and Cairns (Northern QLD) [47]. This was also the case for WNV_KUN_ in an older study [46]. Intraspecies variation may contribute to differences seen in WNV_NSW2011_ transmission in different areas during the outbreak.

Several studies have identified the presence of insect-specific flaviviruses (ISFs) that are maintained in nature in the absence of a vertebrate host [48,49,50,51]. While these viruses have not been associated with disease in humans, mosquitoes persistently infected with ISFs show an altered ability to transmit medically significant viruses, such as WNV [52,53]. In Australia, a novel insect-specific virus named Palm Creek virus, has recently been shown to inhibit the replication of WNV_KUN_ *in vitro* [54]. Therefore, persistent infection of some populations of mosquitoes in Australia with ISFs may affect the transmission of WNV_NSW2011_. This theory needs further investigation.

Historically wading birds, such as herons and egrets have been implicated as the natural reservoirs of WNV_KUN_ [55,56]. However, WNV_NSW2011_ may have adapted to additional bird species common to Australia that have not previously been involved in WNV_KUN_ transmission, such as the house sparrow (*Passer domesticus*), that has been shown to be a competent reservoir for WNV in the USA [57]. The Australian white Ibis (*Threskiornis molucca*) has also been shown to be a reservoir of zoonotic pathogens, including flaviviruses [58] and therefore could have been involved in the transmission of WNV_NSW2011_.

Wild rabbits have been proposed as possible candidates for a cryptic host during the WNV_NSW2011_ outbreak based on observations of large herds of rabbits during 2011 (Peter Kirkland, personal communication). Feral rabbits have been demonstrated to develop a high viraemia following infection with MVEV [59] and Eastern cotton rabbits infected with WNV_NY99_ have been shown to develop sufficient viraemia to infect mosquitoes [60]. Preliminary studies evaluating the rabbit as a potential host of WNV_NSW2011_ have identified a low but sustained viramia in these animals [61]. While a high level of viraemia is generally required for active transmission, studies with Japanese encephalitis virus has demonstrated that despite a low level viraemia in inoculated flying foxes, infection of mosquitoes feeding on these animals still occurred [62]. In addition, differences between feral and lab bred animals and their susceptibility to WNV infection needs to be considered. Another possibility that warrants investigation is that feral animals may already be infected with another microbial agent, which may enhance the transmissibility of WNV_NSW2011_. Other mammals are likely to contribute to the transmission of WNV_NSW2011_. Domestic cats may represent a potential reservoir in urban transmission of WNV_NSW2011_, as the peak viraemia observed in cats infected with WNV_NY99_ may be high enough to infect mosquitoes at low efficiency [63]. A comprehensive evaluation of Australian mammals for the presence of WNV_KUN_-specific antibodies would provide some indication of possible mammalian hosts.

Despite the emergence of a new virulent strain in 2011, virus transmission and disease occurrence did not continue into 2012 and since then only a few equine and human infections have been reported. Reasons for this could be sub-optimal environmental conditions, such as less than average rainfall preventing large populations of *Cx. annulirostris*, pre-existing antibodies to WNV_NSW2011_ or the presence of an undefined vertebrate host (such as the rabbit). An isolate of WNV_KUN_ obtained from mosquitoes collected during 2012 was recently assessed for virulence in an established murine model. The 2012 strain shows similar levels of virulence to WNV_NSW2011_ in mice [64], suggesting that the risk of further disease outbreaks to Australia remains.

Another unusual feature of the Australian outbreak was the absence of severe human cases, unlike what was seen during the on-going North American outbreak. This observation has sparked more questions as to the potential for new strains of WNV_KUN_ to emerge and cause human disease. The emergence of WNV_NSW2011_ demonstrates the changing epidemiology of WNV_KUN_ in Australia from a relatively benign to highly virulent virus and has led to follow-up studies investigating the origin and virulence of WNV_NSW2011_ compared to contemporary and historical strains of WNV_KUN_.

## 4. Evaluation of Virulence of Contemporary and Historical WNV_KUN_ Strains

A collection of WNV_KUN_ strains isolated between 1960 and 2012 were tested for virulence in a young adult mouse model and their full-length genome sequences analysed to identify possible virulence motifs. In an attempt to elucidate the likely origin of the virulent WNV_NSW2011_ strain, isolates from both eastern and western Australia were tested. All five WA isolates were attenuated in the mouse model, whereas two of the strains from eastern Australia showed similar levels of virulence to WNV_NSW2011_. These strains were from the Gulf of Carpentaria (2001) and VIC (1984). These data would suggest that virulent strains of WNV_KUN_ have been circulating in eastern Australia.

WNV_NSW2011_, along with contemporary and historical WNV_KUN_ strains were antigenically typed using a panel of monoclonal antibodies previously shown to differentiate between strains of WNV_KUN_ and other WNVs [5,44,65,66,67,68]. Antigenic analysis confirmed that WNV_NSW2011_ was most closely related to Australian WNV_KUN_ strains and likely emerged from currently circulating strains, rather than the introduction of an exotic WNV strain [2]. The binding profiles of these mAbs to contemporary WNV_KUN_ strains confirmed that all strains tested closely resembled the prototype WNV_KUN_ strain [64]. Interestingly, WNV_KUN_ strains isolated after 2003, including WNV_NSW2011_, were no longer recognised by the mAb that binds the αA3 motif (residues 39–53) in the MTase domain of NS5 [44]. The αA3 motif has not been associated with virulence and this binding pattern may represent an evolutionary marker for WNV_KUN_ strains [64].

Glycosylation of the envelope protein has been associated with neuroinvasion by WNV strains [41]. This was consistent with previous studies that showed attenuated WNV_KUN_ isolates from Australia contained an unglycosylated envelope protein [2,65]. However, the passage history of WNV_KUN_ has since been shown to significantly affect glycosylation, with extensive passaging through cell culture after isolation leading to a change in the glycosylation status of the envelope protein [65]. Indeed, recent analysis of low-passage contemporary WNV_KUN_ strains revealed they all contained glycosylated E protein, regardless of their attenuation status [64]. While glycosylation of the envelope protein and motifs within the NS5 gene are both likely to contribute to the virulence of WNV_NSW2011_ compared to attenuated WNV_KUN_ strains, it is clear that additional viral motifs are responsible for the enhanced virulence of the former. Comparison of full-length genome sequences of historical and contemporary WNV_KUN_ strains will help identify these additional markers of virulence.

## 5. Discussion

Historically WNV_KUN_ has been associated with only mild symptoms in humans and horses. However, under optimal environmental conditions, a newly emerged WNV_KUN_ strain was able to cause an unprecedented outbreak of equine encephalitis in south-eastern Australia. While this outbreak was short-lived, virulent strains of WNV_KUN_ circulate in Australia and will continue to pose a risk to humans and animals. An unexpected feature of the 2011 epidemic was that, despite the large number of equine cases, no severe cases of human disease were reported in areas of intense viral activity. The reasons behind this unusual event could be pre-existing antibodies in the community, a trophism of the virus for equines or minimal contact between humans and infected mosquitoes carrying WNV_NSW2011_. However it seems unlikely that pre-existing antibody against WNV_KUN_ could explain the lack of human cases during the 2011 outbreak since a recent study found a very low prevelance of WNV_KUN_ antibodies in VIC [69]. WNV_NSW2011_ infections was identified in areas in which WNV_KUN_ is generally not associated, especially coastal cites east of the Great Dividing range. This change in epidemiology would suggest that an additional vertebrate host might be involved, which initiated studies into the possibility that rabbits may represent this alternative host. Preliminary studies have identified a low, but sustained viraemia in WNV_NSW2011_-infected rabbits, suggesting these animals may play a role in virus transmission [61].

WNV is estimated to have infected ~4 million humans in the United States (US), causing over 37,000 clinical infections, including 16,196 neuroinvasive disease cases and 1,443 deaths reported to the Centers for Disease Control and Prevention between 1999 and 2012 [70]. For a recent review of WNV focusing on North America and human disease, the reader is referred to Petersen *et al*. [71].

WNV has become endemic in all mainland states of the US, as well as all Canadian provinces since 1999 and is maintained in the US in a bird-mosquito-bird transmission cycle. North American strains of WNV (NA WNV) have been detected in 65 different mosquito species representing 10 different genera [72]. However only a few *Culex* mosquito species are responsible for the majority of transmission including *Cx. pipiens*, *Cx. quinquefasciatus* and *Cx. tarsalis* [71]. *Cx. quinquefasciatus* is also widely distributed in both rural and urban habitats in Australia and is thought to be ornithophilic with bird feeding behavior having been documented [24,73]. *Cx. quinquefasciatus* populations collected in Australia were evaluated and shown to be highly competent for WNV_NY99_ [47]. Therefore, *Cx. quinquefasciatus* could have been involved in urban transmission of WNV_NSW2011_. Other Australian members of the *Culex pipiens* group [74], including *Cx. molestus*, *Cx. australicus* and *Cx. globocoxitus* would also be other valid candidates contributing to transmission of WNV_NSW2011_.

Numerous passerine birds (perching birds of the order *Passeriformes*) develop sufficient viraemia following WNV_NY99_ infection to efficiently infect mosquitoes feeding upon them and therefore are competent amplifying hosts [57]. Furthermore, American crows (*Corvus brachyrhynchos*), blue jays (*Cyanocitta cristata*) and greater sage-grouse (*Centrocercus urophasianus*) completely succumb to infection with a 100% mortality rate [57,75,76]. While the Australian Crow (Little Raven—*Corvus mellori*) was resistant to lethal infection with WNV_NY99_ [43], birds are still likely to play a role in the transmission of WNV_NSW2011_. An evaluation of sera collected from different bird species where extensive WNV_NSW2011_ activity occurred, may provide clues as to particular species that could be involved in virus transmission.

West Nile virus was first reported in the US in 1999 and to date three genotypes belonging to WNV lineage I have been described; NY99, WN02 and SW/WN03. The NY99 genotype includes isolates from human cases from the US epidemic as well as bird and mosquito isolates collected in 1999. The WN02 genotype emerged in 2001, eventually displacing the NY99 genotype and is characterized by an amino acid change from valine to alanine at position 159 of the envelope protein. This amino acid substitution has been associated with earlier transmission and more efficient dissemination in *Cx. pipiens* and *Cx. tarsalis* mosquitoes as compared to the NY99 genotype [77]. However, this phenotype is still under debate [78]. The southwesten genotype, SW/WN03 was first identified in Arizona, Colorado and northern Mexico in 2003 and has subsequently spread to the Upper Texas Gulf Coast region [79]. Furthermore, ongoing dynamic circulation of WNV between the US and Mexico has been demonstrated [80]. While studies have demonstrated the emergence of new genotypes in the US, it is not clear whether these genetic changes will translate into phenotypic changes altering the risk of WNV to the human population. These studies from the US highlight the importance of continual surveillance of WNV to identify changes to the virus genome that could lead to phenotypic changes in the virus. On-going surveillance of WNV_KUN_ isolates in Australia will also provide information as to whether virulent strains continue to circulate, as is the case with a WNV_KUN_ isolate from 2012.

Another important lesson from the North American outbreak was the demonstration that WNV can be transmitted to humans via blood transfusion [81]. The study by Pealer and colleages demonstrated that using blood donor screening, recent donors with a low level viraemia who had not yet developed WNV-specific antibodies could efficiently transmit the infection. In light of these findings, national blood donor screening using nucleic acid tests were initiated in 2003 to ensure transfusion safety [82]. Newly emerging virulent strains of WNV_KUN_ could also pose a similar threat to the safety of the Australian blood supply. Future studies are required to evaluate the risk that newly emerging strains pose to transfusion safety in Australia and whether extensive donor screening is required.

## 6. Conclusions and Future Directions

The epidemiology and ecology of WNV_KUN_ has changed in recent years with WNV_KUN_ no longer a naturally attenuated virus, but one that can and has caused a large outbreak of equine encephalitis and therefore warrants new investigations to evaluate genetic and phenotype changes in currently circulating strains. On-going studies will address the seroprevalence of WNV_KUN_-specific antibodies in humans and virulence motifs within WNV_NSW2011_ that may restrict this virus to equines. Furthermore, future studies need to consider parameters affecting the virus, hosts and vectors separately and in concert in order to better understand risk factors predisposing to disease outbreaks. Further sequence analysis of all contemporary and historical strains of WNV_KUN_ will help elucidate the key residues responsible for enhanced virulence of WNV_NSW2011_. Preliminary studies comparing contemporary, historical and known virulent strains of WNV_KUN_ have identified possible putative virulence determinants and/or evolutionary markers, including the αA3 motif. Given the close genetic relationship between NA WNV strains and WNV_KUN_, results from these ongoing studies will be directly relevant for currently circulating strains in the US. These studies will provide valuable information evaluating the true risk of WNV in a medical and veterinary context.
